# Multi-Scale Analysis of the Damage Evolution of Coal Gangue Coarse Aggregate Concrete after Freeze–Thaw Cycle Based on CT Technology

**DOI:** 10.3390/ma17050975

**Published:** 2024-02-20

**Authors:** Changhao Xin, Yu Yang, Mengze Yang, Junzhen Di, Yidan Sun, Pengfei Liang, Yaohong Wang

**Affiliations:** 1 College of Civil Engineering, Liaoning Technical University, Fuxin 123000, China; 472120637@stu.lntu.edu.cn (C.X.); yangyu9300@163.com (Y.Y.); dijunzhen@126.com (J.D.); 472010038@stu.lntu.edu.cn (P.L.); 2 College of Civil and Marine Engineering, Jiangsu Ocean University, Lianyungang 222000, China; 2020000083@jou.edu.cn; 3 China Railway Fourth Bureau Group Road and Bridge Engineering Co., Ltd., Changchun 130000, China; wangyaohong1@foxmail.com

**Keywords:** coal gangue coarse aggregate concrete, freeze–thaw cycle, compressive strength, X-ray CT, macro–meso combination

## Abstract

This study utilized X-ray computed tomography (CT) technology to analyze the meso-structure of concrete at different replacement rates, using a coal gangue coarse aggregate, after experiencing various freeze–thaw cycles (F-Ts). A predictive model for the degradation of the elastic modulus of Coal Gangue coarse aggregate Concrete (CGC), based on mesoscopic damage, was established to provide an interpretation of the macroscopic mechanical behavior of CGC after F-Ts damage at a mesoscopic scale. It was found that after F-Ts, the compressive strength of concrete, with coal gangue replacement rates of 30%, 60%, and 100%, respectively, decreased by 33.76%, 34.89%, and 42.05% compared with unfrozen specimens. The results indicate that an increase in the coal gangue replacement rate exacerbates the degradation of concrete performance during the F-Ts process. Furthermore, the established predictive formula for elastic modulus degradation closely matches the experimental data, offering a reliable theoretical basis for the durability design of CGC in F-Ts environments.

## 1. Introduction

China is one of the largest coal producers in the world, with a staggering coal output of 4.13 billion tons in 2021, accounting for 52.4% of the global total [1]. However, coal mining also causes environmental problems, one of which is the generation and disposal of a large amount of coal gangue. Coal gangue is a by-product of coal mining and washing, comprising 15–20% of the total coal output [2,3]. It is estimated that China’s current coal gangue volume has exceeded 6 billion tons, covering an area of more than 13,300 ha, and it is still growing at a rate of 500–800 million tons per year [4]. This phenomenon not only wastes many land resources, but it also causes serious environmental problems, such as air pollution, soil erosion, groundwater contamination, etc. [5,6].

With the rapid development of the construction industry, China faces environmental challenges stemming from extensive natural resource extraction. For instance, the current production of concrete necessitates significant quantities of Portland cement as a binder, and large amounts of natural aggregates, leading to substantial environmental degradation during their extraction. To mitigate the environmental impact of the construction sector, researchers have proposed a series of eco-friendly solutions. Some have suggested using additives with active volcanic ash to replace cement during concrete production to reduce cement consumption [7,8,9,10], whereas others suggest modifying concrete using nanomaterials to increase its durability [11,12,13,14]. To reduce the demand for natural aggregates and to mitigate the environmental burden, people have started to study the use of recycled aggregates, industrial waste, and mineral tailings as alternatives to natural aggregates in concrete [15,16,17,18,19]. This alternative not only helps to improve the quality and sustainability of buildings, but it also effectively utilizes waste resources, reduces environmental pollution, and provides a feasible solution for the comprehensive utilization of waste materials such as coal gangue.

Currently, scholars have undertaken extensive endeavors in the field of coal gangue coarse aggregate concrete (CGC) [20]. Through the meticulous examination of coal gangue morphology and rigorous investigations into the mechanical properties and durability of CGC [21], it has been ascertained that coal gangue can be partially substituted for gravel in concrete. However, with the escalation of coal gangue content, the macroscopic performance of concrete typically undergoes a certain degree of impact. The prevailing consensus at present suggests that as the substitution rate of coal gangue increases, the compressive strength of CGC gradually declines. When the replacement rate of coal gangue reaches 100%, the compressive strength of CGC decreases by approximately 15% to 20% [20,22]. However, some studies have also indicated that concrete exhibits improved mechanical properties when coal gangue is used as a substitute for natural aggregates in steel tube concrete [21,23]. Furthermore, employing calcined coal gangue in CGC results in significantly higher compressive strength compared with using non-calcined coal gangue [24]. Examining the influence of coal gangue powder on CGC performance reveals that substituting 10% of cement with coal gangue powder yields the highest compressive strength for CGC [25]. It is noteworthy that notwithstanding coal gangue’s favorable performance under certain specific circumstances, its application in concrete still encounters certain challenges. For instance, the distinctive particle shape and size of coal gangue differs from conventional gravel, whereas the porous structure of coal gangue aggregates results in a higher water absorption rate, consequently diminishing the workability of concrete [22]. Additionally, the presence of adhered impurities, such as coal on coal gangue aggregates, can adversely affect the bonding performance between the cement matrix, leading to a reduction in the durability of coal gangue concrete [26]. Therefore, stringent screening and the pre-treatment of coal gangue are imperative when utilizing it as concrete aggregates to ensure the optimal performance of concrete.

The majority of coal mines in China are located in the cold northern regions. Therefore, considering the savings in terms of the cost of material transportation, the primary application scenario for CGC is in the northern regions. Consequently, it is imperative to consider the significant impact of freeze–thaw cycles (F-Ts) on the mechanical performance and durability of CGC. Extensive research has been conducted on the deterioration patterns of CGC under F-Ts conditions, elucidating the macroscopic mechanical effects of F-Ts on CGC [27,28,29]. Under F-Ts conditions, concrete materials undergo two main changes. Firstly, hydration products of cement expand unevenly, and secondly, continuous phase transitions occur between water and ice. The result of these processes is the continuous amplification of microscopic defects within the concrete, leading to an escalating level of damage, which ultimately causes a gradual decline in the macroscopic mechanical properties of the concrete [30]. The cumulative damage to the microstructural integrity of concrete is a critical factor leading to macroscopic structural failure. Therefore, a comprehensive analysis of the concrete damage caused by F-Ts must be conducted at different scales, and a multiscale mechanism needs to be established. This research direction holds significant importance for a thorough understanding of freeze–thaw damage in concrete, with crucial theoretical implications and practical application value in the prevention and control of engineering disasters in cold regions. Currently, research on CGC under F-Ts conditions primarily focuses on analyzing the evolution patterns of concrete’s mechanical properties at the macroscopic level. Various damage evolution models have been defined based on different performance indicators, predicting the service life of CGC. However, there remains a significant research gap in terms of establishing a quantitative relationship between the microscopic damage and macroscopic mechanical response of CGC and understanding the crack propagation patterns and failure processes under F-Ts conditions.

This study conducted a multi-scale investigation of the F-Ts damage of CGC, based on the characterization approach of “freeze–thaw environment–mesostructure–macroscopic properties”. CGC was selected as the research object, and F-Ts, X-ray computed tomography (CT) scanning, and mechanical property tests were conducted. Digital image processing techniques were applied to extract concrete mesoscopic features and perform three-dimensional reconstruction for visualization. The study conducted a rigorous investigation of the quantitative assessment of mesoscopic damage by analyzing alterations in pore size and the structural morphology in CGC. It quantitatively characterized macroscopic damage based on the initial damage and material property degradation of CGC to establish a connection between macroscopic and mesoscopic damage evolution patterns. This approach provides a novel research method for a comprehensive depiction of freeze–thaw damage in CGC.

## 2. Materials and Methods

### 2.1. Raw Materials

#### 2.1.1. Coal Gangue Coarse Aggregate

**Figure 1:**
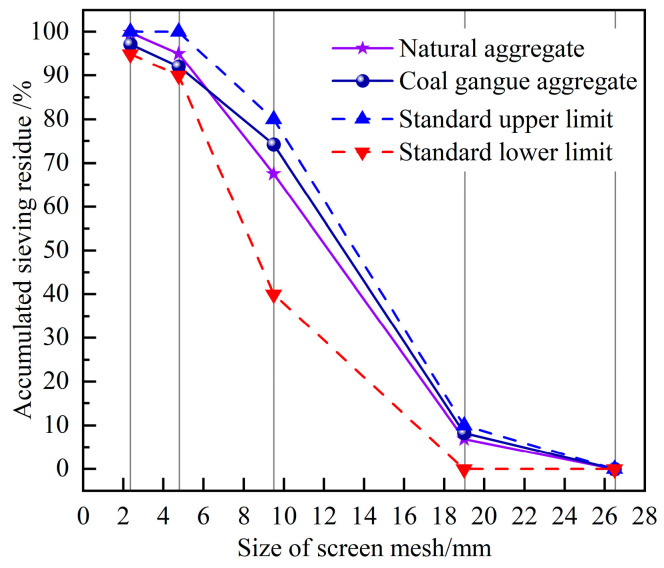
Particle size distribution of aggregates.

The coal gangue coarse aggregate utilized in this study originated from the Hongliulin Coal Mine in Shenmu City, China. To ensure the integrity of test results and to prevent contamination via impurities, such as coal slurry and coal slag, large chunks of coal gangue were manually selected prior to the experiment. After the selection process, the coal gangue was crushed and screened using a jaw crusher. Then, it was mixed using a precise ratio to create coarse aggregates of coal gangue with particle sizes ranging from 5 to 20 mm. Figure 1 illustrates the distribution of particle sizes. It is evident that the particle size distribution of the coal gangue aggregates used in this study meets the requirements for coarse aggregate particle size distribution outlined in the Chinese standard, JGJ 52-2006 [31].

The coal gangue coarse aggregates were evaluated for their basic characteristics, in accordance with the guidelines provided in GB/T 14685-2011 [32], and the findings are tabulated in Table 1. Physical and mechanical performance indicators of the coarse aggregates include apparent density and water absorption to reflect the quantity of internal closed and open pores within the aggregate [33]. The crushing index primarily indicates the compressive strength of the aggregate. These three indicators are standard metrics for evaluating the quality of coarse aggregate materials. Put differently, higher apparent density, lower water absorption, and a lower crushing index signify superior material properties of coarse aggregates, rendering them more suitable for use in concrete. An examination of Table 1 reveals that, with similar particle size distributions, coal gangue coarse aggregates exhibit water absorption at a significantly higher rate than that of natural coarse aggregates, whereas the remaining indicators are relatively close to those of natural coarse aggregates. Therefore, coal gangue coarse aggregates can be deemed a feasible alternative to natural aggregates. However, it is important to note that the porous and water-absorbing characteristics of coal gangue can also result in the poor workability of concrete containing coal gangue aggregate [6,34]. Pre-wetting the aggregate can prevent issues such as difficulty in mixing and compacting fresh concrete, as well as excessive internal porosity caused by water absorption. Moreover, the water retention properties of coal gangue aggregate can mimic the effects of internal curing, which is observed in lightweight aggregates [35]. Therefore, pre-wetting the aggregate stands as one of the more effective measures to enhance the workability of concrete when incorporating coal gangue aggregate.

#### 2.1.2. Other Materials

Jidong Cement Co., Ltd. (Tangshan City, China) provided the ordinary Portland cement (P·O 42.5) used in the conducted experiment. The natural coarse aggregates, consisting of crushed stone, exhibited a consistent gradation, resembling that of the coal gangue coarse aggregates. The distribution of particle sizes is depicted in Figure 1. Natural river sand, with a fineness modulus of 2.85, was employed as the fine aggregate. The water-reducing agent employed was a polycarboxylate superplasticizer with a water-reducing rate of 25%. The mixing water utilized was obtained from Fuxin City municipal tap water.

### 2.2. Specimen Preparation

The experiment focused on the coal gangue replacement rate as a variable, investigating four volume-based replacement rates (r = 0%, 30%, 60%, 100%), where coal gangue substitutes natural coarse aggregates. Concrete mix proportions for each group were determined using the volumetric method, based on the mix design calculation outlined in the Chinese standard, JGJ 55-2011 [36], detailed in Table 2. In this study, the designated strength grade for the reference concrete was set at C40. Before the experiment, coarse aggregates were pre-wetted by sprinkling water on them to achieve a saturated surface-dry state. The water values in the table indicate the net water quantity.

**Figure 2:**
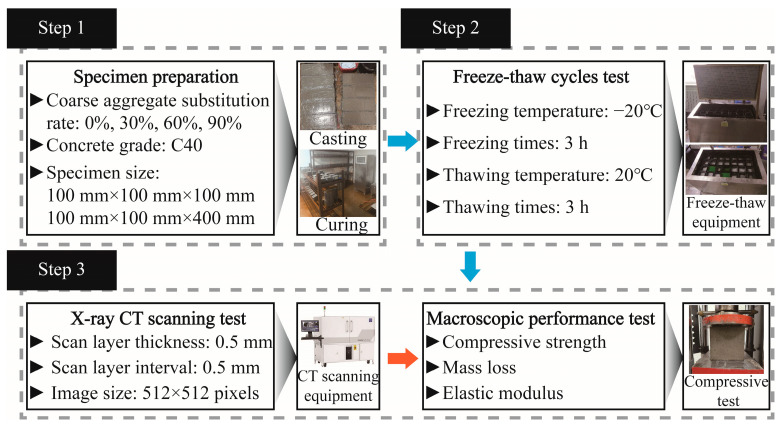
Overall research program flow.

In accordance with the Chinese standard, GB/T 50081-2019 [37], when the maximum aggregate size in concrete is 31.50 mm, the minimum cross-sectional dimensions for specimens in tests, such as uniaxial compression should be 100 mm × 100 mm. Considering that the maximum aggregate size in our study’s concrete was 26.50 mm, the selected cubic specimen dimensions of 100 mm × 100 mm × 100 mm were chosen using this requirement. Additionally, prism specimens measuring 100 mm × 100 mm × 400 mm were also fabricated. Following demolding, the specimens were placed in a standard curing room with a temperature of (20 ± 2) °C and a relative humidity of 95%, where it underwent curing for 28 days. The density of concrete in this study varied depending on the coal gangue replacement rate, as follows: 2451 kg/m^3^; for 0%, 2437 kg/m^3^; for 30%, 2424 kg/m^3^; for 60%, and 2407 kg/m^3^; for 100%. These values provide insights into the impact of coal gangue on the overall density of the concrete mixtures. The experimental process of this study is shown in Figure 2.

### 2.3. Experiment Methods

#### 2.3.1. Freeze–Thaw Cycles Test

**Figure 3:**
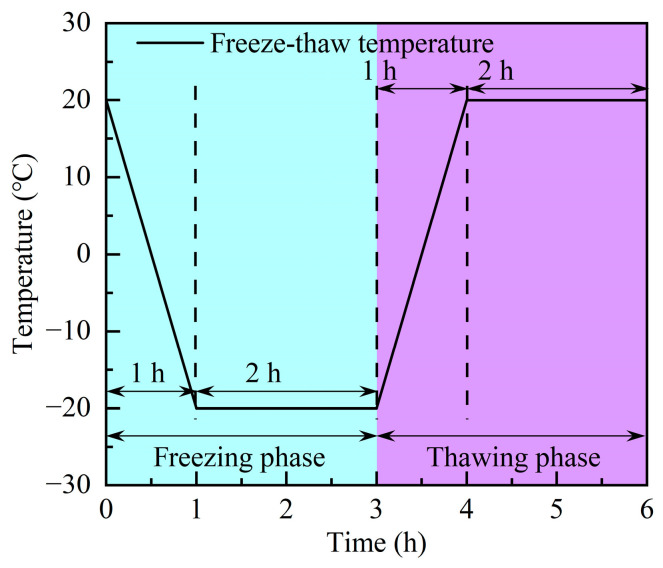
Freeze–thaw cycle with temperature–time.

The concrete F-Ts test employed the KDR-V9 rapid F-Ts test machine, to adhere to the rapid F-Ts method specified in the Chinese standard, GB/T 50082-2009 [38]. The experimental setup entailed a gradual reduction in temperature, from 20 °C to −20 °C, followed by an ascent back to 20 °C; this constituted one complete F-T, with each cycle spanning approximately 6 h. The temperature–time variation curve during the F-Ts is illustrated in Figure 3. For the GC60 and GC100 groups, three specimens were retrieved after every 5 F-Ts, they were subjected to surface moisture removal by wiping, and their mass loss was quantified by utilizing an electronic scale. Conversely, for the GC0 and GC30 groups, mass loss assessments were conducted following every 10 F-Ts. When the mass loss surpassed 5%, this was indicative of concrete damage, and the test was terminated. A total of 186 specimens underwent testing in this experiment. The NM-4B non-metal ultrasonic testing analyzer was employed to assess the elastic modulus loss of the specimens. Furthermore, uniaxial compressive strength tests were performed using the TAW-2000 (Chang Chun City Chao Yang Test Instrument Co., Ltd., Changchun, China) universal servo testing machine.

#### 2.3.2. X-ray CT Scanning Test

To comparatively analyze microstructure changes in specimens that were subjected to the same conditions, a chosen specimen underwent CT scanning experiments using the ZEISS Xradia Context industrial CT machine (Carl Zeiss AG, Oberkochen, Germany) across various F-Ts counts. A dual helical CT scanning method was employed, with a maximum tube power of 320 W, a focus-to-detector distance of 805 mm, a focus-to-object distance of 110 mm, and an integration time of 2000 ms. During CT scanning, the specimen, post F-Ts, underwent axial scanning with a scan layer thickness and interval of 0.5 mm. Following each scan, a total of 200 two-dimensional cross-sectional scan images, each with dimensions of 512 × 512 pixels, were acquired. Subsequently, the CT data were imported into the Avizo software (version number: 2022.2) for threshold segmentation, three-dimensional reconstruction, and computation of pore structure characteristics.

## 3. Results and Discussion

### 3.1. Analysis of the Relative Dynamic Modulus of Elasticity and Mass Change

**Figure 4:**
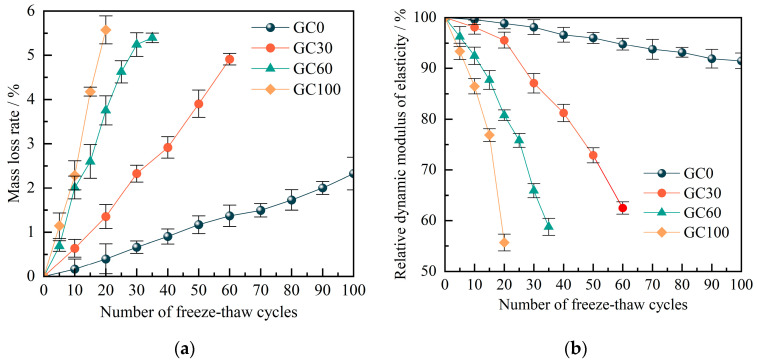
Curves of (**a**) the mass loss rate and (**b**) relative dynamic elastic modulus with freezing and thawing numbers.

Figure 4a shows the distribution characteristics of the rate of mass loss (*M*_loss_) in concrete after F-Ts underwent different coal gangue coarse aggregate substitution rates. The *M*_loss_ of concrete exhibits a substantial dependence on the substitution rate of coal gangue. In the absence of coal gangue additions, the *M*_loss_ of concrete demonstrates a less pronounced increase with the number of F-Ts, with the *M*_loss_ of the GC0 group reaching only 2% after 100 F-Ts. Conversely, the *M*_loss_ of GC30, GC60, and GC100, which represent concrete samples with coal gangue as a substitute for natural coarse aggregate, is notably higher than that of the GC0 group. As the number of F-Ts increases, the mass of different CGC samples gradually decreases due to the spalling of surface mortar. Furthermore, the freeze–thaw damage of CGC becomes more severe with increases in coal gangue content, leading to a corresponding increase in mass loss. More specifically, when the coal gangue replacement rates are 30%, 60%, and 100%, the F-Ts thresholds at which the *M*_loss_ of concrete exceeds 5% are 60, 30, and 20, respectively. This suggests that the substitution rate of coal gangue exerts a controlling influence on the frost resistance of concrete. The addition of coal gangue accelerates the spalling of the mortar on the concrete surface, leading to more pronounced loss in mass. Hence, it is evident that the utilization of CGC in damp and frigid regions will significantly reduce the lifespan of concrete.

The relative dynamic elastic modulus (*E*_r_) of CGC that is subjected to F-Ts is illustrated in Figure 4b. For CGC with 0% coal gangue content, the *E*_r_ loss rates are 1.1%, 5.2%, and 8.6% at F-Ts numbers of 20, 60, and 100, respectively. In the case of 30% coal gangue content, the *E*_r_ loss rates for CGC are 4.4% and 37.5% at 20 and 60 F-Ts, respectively. Notably, for 60% and 100% coal gangue content, the *E*_r_ loss rate of CGC is more than 40% when the number of F-Ts is 35 and 20, respectively. This trend means that when higher coal gangue content correlates with increased *E*_r_ loss, and for content exceeding 60%, a sharp escalation in *E*_r_ loss is observed.

This phenomenon can be attributed to the elevated generation of internal cracks and pores within the concrete as the coal gangue content rises. The rapid infiltration of water under external pressure results in a volumetric expansion of water by up to 9% post-freezing. The combined effects of expansion pressure and osmotic pressure intensify the propagation and interconnections between concrete cracks, leading to the emergence of new micro-cracks. As the number of F-Ts rises, there is a corresponding increase in both the quantity and size of microcracks within the concrete. This leads to heightened internal damage and a significant reduction in the *E*_r_.

### 3.2. Relationship between Compressive Strength and Cycle Times

**Figure 5:**
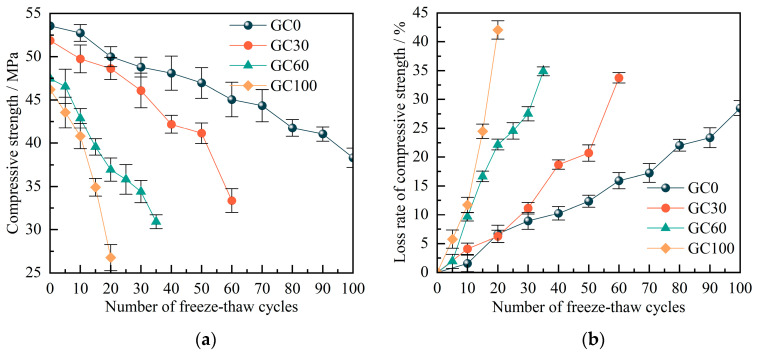
Curves of (**a**) compressive strength and (**b**) the loss rate of compressive strength with freezing and thawing numbers.

Regarding the F-Ts environment, the change law of the compressive strength (*σ*_c_) of CGC is depicted in Figure 5. It can be observed that the CGC exhibits a significant disparity in initial *σ*_c_, ranging from approximately 45 MPa to around 55 MPa. Therefore, when analyzing the deterioration of CGC performance in F-Ts environments, it is inadequate to merely compare the numerical values of *σ*_c_. A more comprehensive analysis of *σ*_c_ loss rates is also necessary.

When the concrete experienced a *M*_loss_ exceeding 5%, the *σ*_c_ values for substitution rates of 30%, 60%, and 100% coal gangue were 33.36 MPa, 30.91 MPa, and 26.79 MPa, respectively. Compared with the unfreeze–thawed specimens, the *σ*_c_ loss rate was 33.8%, 34.9% and 42.1%, respectively. This observation underscores that as the coal gangue replacement rate increases, the *σ*_c_ of CGC diminishes, and it is accompanied by an escalating loss rate. This phenomenon can be attributed primarily to the inferior strength of coal gangue compared with conventional aggregate. Additionally, the compactness of CGC is inferior to that of standard concrete, with compactness being a pivotal factor influencing concrete strength. Moreover, the porous and water-absorbing characteristics of coal gangue significantly influence the capillary water absorption of CGC [33]. In the same freeze–thaw environment, the capillary water absorption capacity of CGC surpasses that of ordinary concrete, with water absorption increasing as the coal gangue content rises. The high water absorption rate of coal gangue aggregate results in a reduction in the hydration degree of cement in the interface transition zone (ITZ) between the aggregate and concrete matrix, thus severely constraining the freeze–thaw resistance of CGC [39]. Furthermore, the strength of CGC exhibited a declining trend with an increase in F-Ts. This is because with the progression of F-Ts, the frost heave stress makes the internal cracks of CGC gradually deepen, and even run through, resulting in a general reduction in the *σ*_c_ of CGC.

### 3.3. Analysis of Meso-Characteristics of Coal Gangue Concrete

#### 3.3.1. Two-Dimensional Pore Structure of Coal Gangue Concrete under Freeze–Thaw Cycle

**Figure 6:**
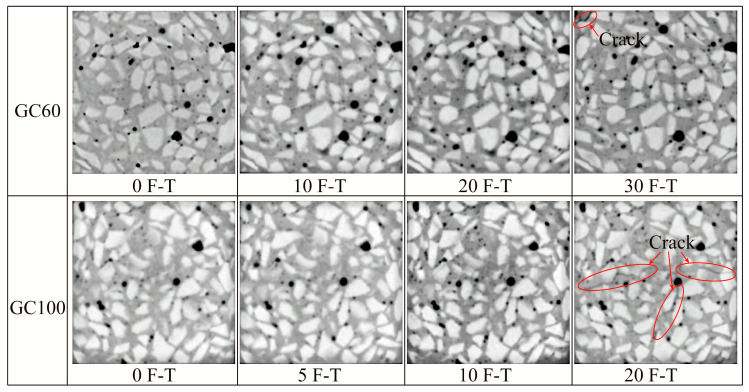
Gray images of the axial CT scanning section of concrete after different freeze–thaw cycles.

To further explore the mechanisms behind the degradation of mechanical properties in CGC during the F-Ts, X-ray CT technology was employed to study the microstructural changes. Due to space limitations, this text presents only the grayscale images of the same scanned section of concrete samples, with coal gangue substitution rates of 60% and 100%, following various F-Ts (as depicted in Figure 6). As the number of F-Ts increased, it was observed that the specimens exhibited a gradual increase in the number of pores, along with an increase in pore sizes. At the end of the F-Ts for all groups of specimens, distinct cracks appeared inside the concrete, with a greater number of cracks observed in specimens with higher coal gangue substitution rates. After undergoing 30 F-Ts, the concrete exhibited only a short crack when the proportion of coal gangue replacement reached 60%. However, at a coal gangue replacement rate of 100%, internal cracks in the concrete specimens appeared and penetrated the entire cross-section after only 20 F-Ts, leading to a sharp decline in the concrete’s performance. The results of the CT scans further validate the experimental results concerning the concrete’s compressive strength, as discussed in Section 3.2.

**Figure 7:**
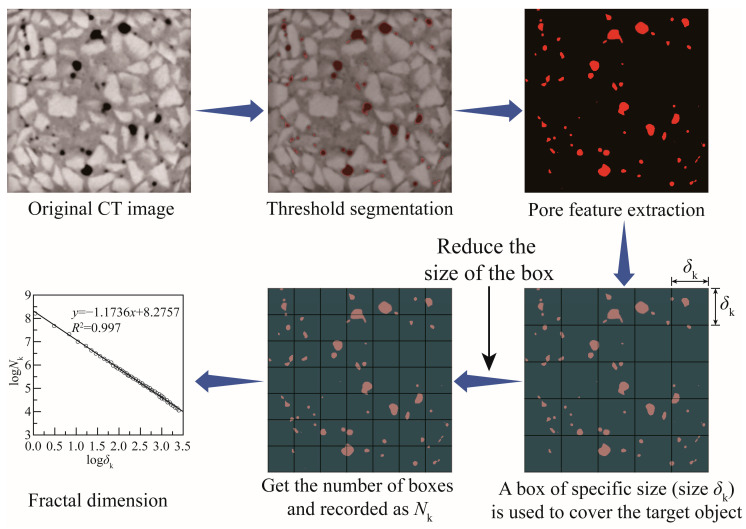
Calculation process of the fractal dimension of coal gangue concrete.

Pores in concrete typically exhibit irregular and disordered shapes, deviating from the assumed smoothness. Across all scales, materials exhibit fractal characteristics in terms of their pore shape, area, and volume [40]. To conduct a more in-depth quantitative analysis of the pore characteristics in CGC, this section employs a fractal dimension (*D*) to characterize the distribution of pore sizes. The study employed the box-counting method, as depicted in Figure 7, to develop a volume-based fractal model for simulating the material’s fractal structure. Initially, pores within the concrete specimen were covered using boxes of side length *δ*_k_, and the number of boxes containing pores, denoted as *N*_k_, was recorded. Subsequently, the size of the boxes was systematically reduced, and the corresponding number of boxes was determined. Finally, a linear regression analysis was performed utilizing double-logarithmic coordinates of the recorded box counts and sizes, whereby the slope represents the box dimension. The equation is expressed as follows:

*D* = log*N*_k_/log(1/*δ*_k_), (1)

**Figure 8:**
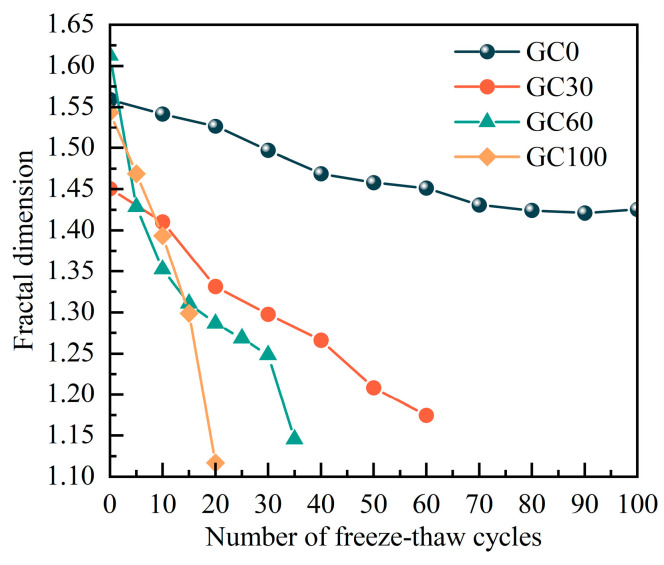
Curves of the fractal dimension with the freeze–thaw number.

The fractal dimension serves to characterize the complexity of the pore structure in concrete. A higher fractal dimension signifies the coexistence of numerous pores with varied sizes, whereas a lower fractal dimension indicates a more uniform pore structure with increased connectivity of smaller pores. Figure 8 illustrates the outcomes of implementing the box-counting technique on various samples, showcasing the fractal dimension of their pores. Although the initial fractal dimensions of the four groups of specimens differ significantly, indicating varying degrees of complexity in pore structures, all groups exhibit a decreasing trend, in terms of fractal dimension, with the progression of F-Ts. After comparing groups GC30, GC60, and GC100, their fractal dimensions were nearly identical at the conclusion of the F-Ts. The reason for this convergence is that, in the initial phases of F-Ts, the internal formations of tiny cavities in the samples are uneven and jagged. However, as the F-Ts progress, the water in the pores undergoes continuous phase transitions between liquid and solid, gradually enlarging the initially formed small pores and increasing the proportion of large pores. The distribution of pore sizes becomes simplified. Simultaneously, the pore walls inside the concrete undergo smoothing due to the frictional effect of water, resulting in more regular shapes.

The graph illustrates that the final fractal dimensions of the GC0, GC30, GC60, and GC100 specimens decreased by 8.6%, 19.0%, 22.6%, and 27.7%, respectively, in comparison with their initial fractal dimensions. This indicates that as the coal gangue substitution rate increases, the concrete’s fractal dimension decreases more, signifying a higher proportion of large pores within the specimens. This observation aligns with the pattern of alterations in the comparative dynamic elastic modulus that was discussed in Section 3.1. More specifically, using the same F-Ts count, higher coal gangue replacement rates resulted in lower compactness within the concrete specimens. Consequently, under the influence of F-Ts, the connectivity of internal pores acceleratef, leading to a larger proportion of large-sized pores. In summary, a higher coal gangue replacement rate corresponds to a more profound degradation of concrete under F-Ts conditions.

#### 3.3.2. Three-Dimensional Pore Structure of Coal Gangue Concrete in a Freeze–Thaw Cycle

**Figure 9:**
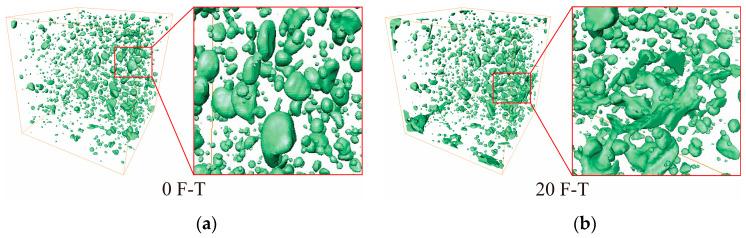
The pore structure in three dimensions during (**a**) 0 and (**b**) 20 freeze–thaw cycles for GC100. The red box magnified area shows the connectivity of concrete pores under different F-Ts.

To obtain a more precise depiction of the spatial distribution features and changes in the pore structure of CGC during the F-Ts procedure, and to quantitatively describe mesoscopic damage and degradation patterns, the acquired two-dimensional images underwent three-dimensional reconstruction for visual analysis. The two-dimensional slice data were transformed into three-dimensional volumetric data, establishing a three-dimensional pore model that vividly depicts the morphological characteristics of the internal pore structure in coal gangue, as illustrated in Figure 9 (demonstrated here as a replacement rate of 100% with coal gangue). The pore structure is represented by the green color in the diagram. Significant changes in the internal pore structure of CGC can be observed from the figure’s three-dimensional pore structure, prior to experiencing macroscopic damage. As the number of F-Ts increases, the pores continue to multiply. With 0 F-Ts, the pores exhibit smaller and more independent distributions. After the F-Ts are completed, certain pores link together, and the distribution is primarily controlled by interconnected pores.

**Figure 10:**
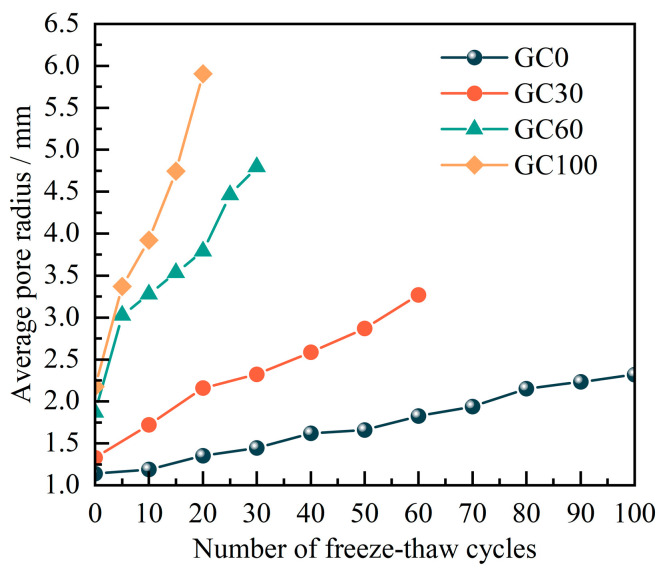
Curves of the average pore radius with freeze–thaw numbers.

To precisely measure the progress of the entire pore formation in CGC and examine the mesoscopic patterns of damage and degradation, an individual analysis was performed on each pore. Methods were employed to characterize the pores by tallying the number of voxels representing pores and the number of pixels outlining pore boundaries, enabling the determination of pore volume and surface area. The average pore radius in the specimens was statistically determined (see Figure 10). The statistical results indicate that, during the F-Ts process, the average radius of pores in CGC increases with the number of F-Ts. At the end of the F-Ts, the final average pore radius of the GC0, GC30, GC60, and GC100 specimens increased by 103.2%, 145.9%, 156.6%, and 171.5%, respectively, compared with their initial states. This increase is mainly attributed to the expansion and damage of small-sized pores and their connections, aligning with the analysis of the fractal dimension in the study of two-dimensional pore structures under F-Ts conditions.

### 3.4. Evolution Law of the Freeze–Thaw Damage of Coal Gangue Concrete

Macroscopic damage mechanics adopt an approach wherein damage is treated as a continuous distribution at the macro level [41,42,43,44]. It phenomenologically captures material damage characteristics by employing macroscopic damage variables. However, this framework does not delve into the intricate physical mechanisms responsible for material deformation and failure. In contrast, mesoscopic damage mechanics focus on the examination of shapes, patterns, and the developmental traits of diverse types of damage at the internal particle and microcrack levels [45,46,47,48,49]. This mesoscale perspective provides a more detailed understanding of the underlying processes contributing to material damage, offering insights into the microstructural intricacies governing deformation and failure mechanisms [50]. In this study, two different scales were chosen for measuring damage, and diverse metrics were selected for characterizing damage. By conducting cross-scale damage identification and comparative studies on the same object, a method was proposed for defining damage variables that comprehensively consider mesoscopic structural information. The objective of this method was to investigate the correlation between mesoscopic data and the macroscopic mechanical behavior of substances.

#### 3.4.1. Definition of the Macro–Meso Freeze–Thaw Damage Variable

Kachanov damage theory assumes that the deterioration process of materials involves a reduction in the effective load-bearing area due to the existence of microdefects. This gradual phenomenon eventually leads to the deterioration of the material. Regarding the meso-feature extracted in this paper, the concrete porosity was defined as the ratio of the internal failure zone volume to the nominal bearing volume of concrete. Hence, the meso freeze–thaw damage variable (*S*_n_) of CGC, calculated using the conventional method, is as follows [51,52]:

(2) $$S_{n}=A/\tilde{A},$$

where *A* is the nominal bearing volume of concrete, and $\tilde{A}$ is the internal failure zone volume of concrete.

In accordance with the macroscopic phenomenological theory, the degradation and attenuation of concrete’s mechanical properties are perceived as the macroscopic manifestation of the progressive accumulation of damage. The response exhibited by macroscopic mechanical properties serves as an indicator to characterize the extent of concrete’s damage deterioration. Traditional macro freeze–thaw damage variables (*S*_n_′) for concrete are typically represented through alterations in the elastic modulus of the material, as follows [53]:

*S*_n_′ = 1 − (*E*_n_*/E*_0_), (3)

The elastic modulus of saturated unfreeze–thawed CGC is represented by *E*_0_, whereas *E*_n_ represents the elastic modulus of CGC after undergoing various F-Ts.

**Figure 11:**
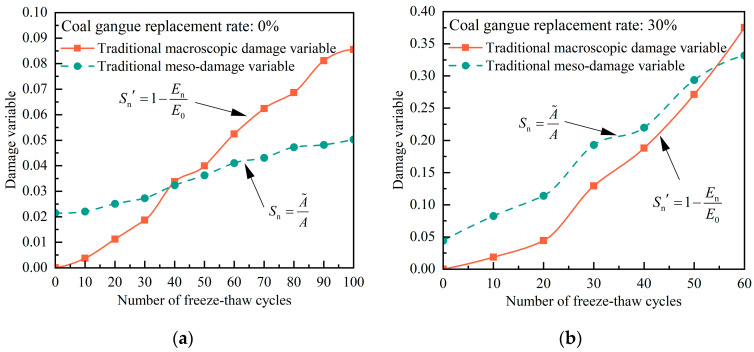

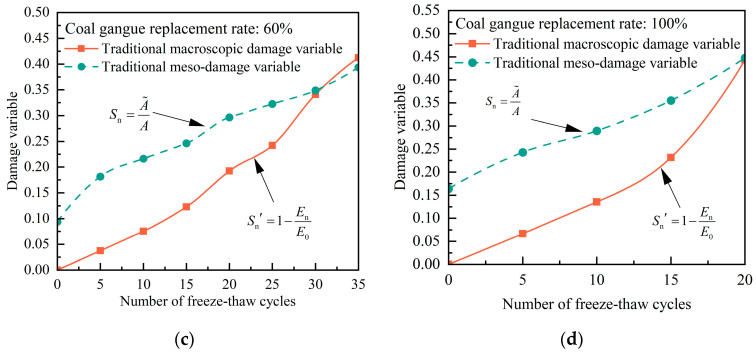
Macro and meso freeze–thaw damage evolution of concrete at (**a**) 0%, (**b**) 30%, (**c**) 60%, and (**d**) 100% coal gangue; substitution plots defined by traditional methods.

Entering the experimental data into Equations (2) and (3) yields conventional macroscopic and mesoscopic damage evolution curves in different F-Ts, as depicted in Figure 11. It is evident that the macroscopic and mesoscopic damage variables of CGC increase with an increase in the number of F-Ts, although there are significant differences between the evolution paths of damage. The macroscopic damage variable, defined by traditional methods, assumes an initial damage value of zero, neglecting the initial defects in the concrete. Mesoscopic damage variables exhibit relatively small damage values and evolution rates.

The decline in the physical–mechanical characteristics of concrete occurs due to the combined effects of a reduction in the functional load-carrying region and alterations in the morphology of pore structures. To define damage variables, it is imperative to consider two physical mechanisms of freeze–thaw damage. The variation in concrete porosity serves as a precise indicator of the impact, arising from the reduction in the functional load-bearing area, of the internal damage incurred by concrete during F-Ts. Calculating the fractal dimension of the pores facilitates the assessment of structural changes in the concrete pore system following F-Ts. The fractal dimension of the pores exhibits a positive correlation with the degree of concrete damage after F-Ts. More specifically, a higher fractal dimension signifies a more intricate pore structure during the F-Ts process, leading to intensified stress concentration in the concrete, and consequently, more severe damage. Moreover, incorporating pore fractal dimensions with values exceeding 1 into a novel formula for modifying mesoscopic damage variables, grounded in porosity definition, provides a direct elucidation of the relationship between pore fractal dimensions and concrete damage post F-Ts. This approach takes into account an additional physical mechanism of damage. Consequently, this study, by considering both porosity and the fractal dimension of pores, establishes novel mesoscopic variables for freeze–thaw damage in CGC, thereby rectifying mesoscopic damage variables for CGC subjected to F-Ts. The modified mesoscopic damage variable (*S*_Tn_) is expressed as follows:

(4) $$S_{\mathrm{Tn}}=S_{n}D_{n}=(A/\tilde{A})(-\lim(\ln N_{k}/\ln\delta_{k})),$$

where, *S*_Tn_ is the modified meso-damage variable of freeze–thawed concrete, and *D*_n_ is the fractal dimension of concrete pores after being subjected to different F-Ts times.

Given that the traditional macro freeze–thaw damage variable typically begins with a value of 0, the initial defects in concrete must be considered to achieve a more precise determination of the macroscopic damage variable after F-Ts; it is necessary to establish an elastic modulus of concrete that is devoid of initial defects (*E*). However, in practice, nearly all concrete contains some initial defects, making it challenging to directly measure the value of *E* using conventional testing methods. In this study, the *E* is inferred through a reverse engineering approach that considers the damage characteristics of fully saturated and unfrozen concrete. The primary methodology involves incorporating the mesoscopic freeze–thaw damage variable into the calculation of the elastic modulus of concrete, thereby mitigating the influence of initial defects and enabling the calculation of the elastic modulus for undamaged concrete.

*E* = *E*_0_/(1 − *D*_0_*η*_0_), (5)

where *η*_0_, *D*_0_ and *E*_0_ are the porosity, pore fractal dimension, and elastic modulus of concrete after undergoing 0 F-Ts, respectively.

The macroscopic damage variable *S*_En_ for freeze–thawed concrete, corrected based on the undamaged elastic modulus, can be expressed as:

*S*_En_ = 1 − (*E*_n_*/E*), (6)

**Figure 12:**
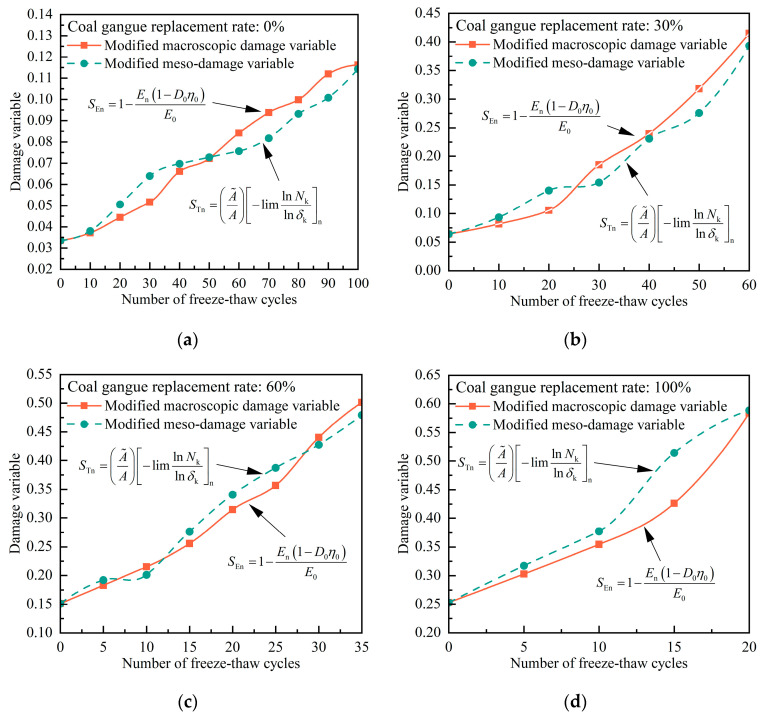
Macro and meso freeze–thaw damage evolution of concrete at (**a**) 0%, (**b**) 30%, (**c**) 60%. and (**d**) 100% coal gangue; substitution plots defined by modified methods.

Upon inputting the test data into the aforementioned equation, the calculated evolution curves for the modified macro and meso freeze–thaw damage variables can be obtained. The corresponding results are illustrated in Figure 12. The examination of Figure 10 reveals that the evolution pattern of the meso-damage variable, based on the definition of pore size and structural shape, closely corresponds with that of the macroscopic damage variable, considering the initial defects in concrete. This coherence proves beneficial for maintaining uniformity in terms of damage identification across different scales of the same material.

#### 3.4.2. Prediction of Deterioration Law of Elastic Modulus Based on Meso-Damage

To conduct an in-depth examination of the impact of meso-structure on the macroscopic mechanical properties of CGC following F-Ts, it is essential to develop a model that articulates the relationship between elastic modulus attenuation and meso-damage variables that are specific to CGC under freeze–thaw conditions. The established macroscopic and mesoscopic damage evolution patterns, rooted in mesoscopic features, demonstrate a noteworthy consistency, and they comprehensively address the uncertainty associated with mesoscopic structural characteristic parameters in freeze–thawed concrete. Through the amalgamation of Equations (4) and (6), the ensuing relationship between microscopic damage variables and elastic modulus can be derived as follows:

*E*_n_′ = (1 – *S*_Tn_)*E*, (7)

where *E*_n_′ is the predicted value of the elastic modulus.

**Figure 13:**
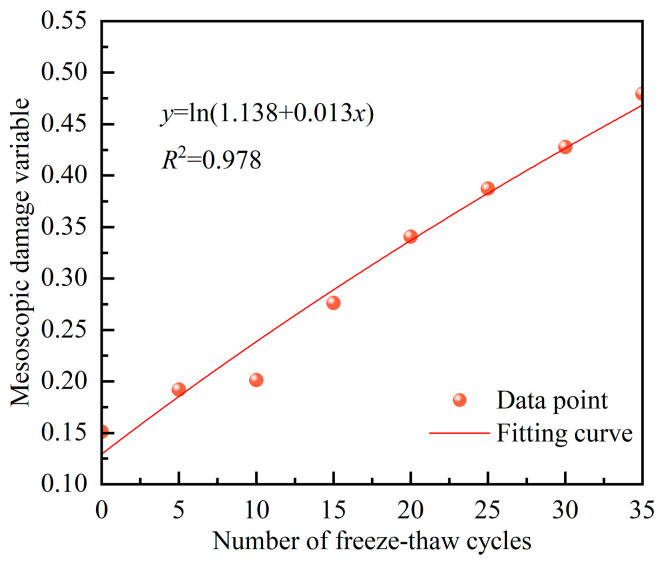
The variation curve of the mesoscopic damage variable with the number of F-Ts.

This investigation employs concrete with a 60% substitution of coal gangue as an illustrative case. Utilizing Equation (4), the values of mesoscopic damage variables for various F-Ts were calculated, yielding a predictive, yet straightforward, formula for the degradation of the elastic modulus. Utilizing the function form, *y* = ln(*a* + *bx*), to characterize the non-linear features of mesoscopic damage, where material parameters a and b can represent the extent of damage during a single F-Ts. Figure 13 illustrates the relationship curve between mesoscopic damage variables and the number of F-Ts.

Substituting the mesoscopic damage variable function from Figure 13 into Equation (7), the predicted formula for elastic modulus degradation based on microscopic damage is obtained as follows:

*E*_n_′ = (1 − ln(1.138 + 0.013*n*))*E*, (8)

The swift prediction of the freeze–thawed concrete elastic modulus is attainable through the utilization of Equation (8), and the resultant predicted values can be compared with experimentally measured data values, as outlined in Table 3. Upon scrutinizing the data in Table 3, it is apparent that the predicted values closely align with the measured values, signifying the capability of the predicted results to mirror the degradation trend of the elastic modulus. This affirms the feasibility of employing mesoscopic damage prediction for forecasting the degradation of the elastic modulus in freeze–thawed concrete. The model quantitatively characterizes the correlation between mesoscopic structural features and concrete mechanical parameters, establishing crucial conditions for developing a multiscale damage model. It integrates the behavior of concrete’s internal components with its macroscopic mechanical behavior. Furthermore, it establishes a theoretical foundation for conducting mechanical property experiments through numerical models in the future, which carry significant practical implications.

## 4. Conclusions

Focusing on CGC, this study conducted F-Ts experiments and CT scanning tests using various replacement rates of coal gangue aggregate. Utilizing digital image processing techniques, the mesoscopic features of CGC after F-Ts were extracted. Subsequently, a predictive model for the mesoscale freeze–thaw damage variables and elastic modulus of CGC was established. The conclusions drawn from this investigation are as follows:

1. With an increase in the replacement rate of coal gangue aggregate in freeze–thaw environments, the frost resistance of concrete gradually diminishes. In particular, when the coal gangue replacement rate exceeds 60%, the performance of concrete deteriorates sharply during freeze–thaw cycles. Therefore, under cold and humid climate conditions, choosing coal gangue as a replacement for gravel in concrete may significantly shorten the service life of concrete. Hence, in cold regions, the replacement rate of coal gangue aggregate should not exceed 30% when substituting natural aggregates.
2. Conventional methods, if the initial defects in concrete and the impacts of meso-structural changes on damage expansion have been neglected, exhibit noticeable disparities in the evolution curves of macroscopic and mesoscopic damage variables. The macroscopic and mesoscopic freeze–thaw damage variables proposed in this study, accounting for pore size and shape, facilitate the cohesive identification of damage in the same material across different scales. This approach introduces a novel quantitative method for the multi-scale investigation of damage characteristics in CGC subjected to F-Ts.
3. The prediction model for the elastic modulus of CGC under various F-Ts provides a means to assess CGC damage by systematically examining the diverse influences of pore size and pore structure. It can quantitatively describe the evolution of microstructure and macroscopic mechanical response.

## 5. Futures and Perspectives

This study provides new insights and predictive tools for understanding the performance variations of CGC in F-Ts environments. However, there are still numerous future research directions worthy of exploration. For instance, the further refinement of multiscale analysis methods and enhancements for predictive models may incorporate the influences of additional concrete material characteristics and environmental conditions. Moreover, exploring new digital image processing techniques and numerical simulation methods can yield more accurate and clear microscopic structural data regarding concrete, thereby improving the precision of predictive models.

## Figures and Tables

**Table 1:** Basic properties of coal gangue coarse aggregate and natural aggregate.

Classification	Apparent Density (kg/m^3^)	Packing Density (kg/m^3^)	Crushing Index (%)	Water Absorption Rate (%)
Coal gangue coarse aggregate	2560	1360	12	5.3
Natural coarse aggregate	2650	1620	10	0.4
Natural fine aggregate	2780	1430	-	0.2

**Table 2:** Mix proportion design of coal gangue concrete (kg/m^3^).

Group	Cement	Water	Natural Aggregate	Coal Gangue Aggregate	Sand	Water Reducer
GC0	367	165	1291	0	637	3.7
GC3	367	165	904	374	637	3.7
GC6	367	165	517	748	637	3.7
GC10	367	165	0	1247	637	3.7

**Table 3:** Measured and predicted values of the elastic modulus of concrete when coal gangue content is 60%.

Number of F-Ts	Measured Value of Elastic Modulus [GPa]	Predicted Value of Elasticity Modulus [GPa]	Relative Error
0	3.13	3.21	2.5%
5	3.01	3.00	0.3%
10	2.89	2.81	2.8%
15	2.75	2.63	4.5%
20	2.53	2.45	3.2%
25	2.37	2.28	3.9%
30	2.06	2.12	2.9%
35	1.84	1.97	6.8%

## Data Availability

Data are contained within the article.

## Abbreviations

*M*_loss_	rate of mass loss
*E*_r_	relative dynamic elastic modulus
*σ*_c_	compressive strength
*D*	fractal dimension
*N*_k_	number of boxes
*δ*_k_	size of the box
*S*_n_	the traditional meso freeze–thaw damage variables
*S*_n_′	the traditional macro freeze–thaw damage variables
*A*	the nominal bearing volume of concrete
*Ã*	the internal failure zone volume of concrete
*E*_0_	the elastic modulus of saturated unfreeze–thawed CGC
*E*_n_	the elastic modulus of CGC after undergoing various F-Ts
*S*_Tn_	the modified meso freeze–thaw damage variable
*D*_n_	the pore fractal dimension of concrete under different F-Ts times
*η*_0_	the porosity of concrete after undergoing 0 F-T
*D*_0_	the pore fractal dimension of concrete after undergoing 0 F-T
*S*_En_	the modified macro freeze–thaw damage variable
*E*_n_′	the predicted value of elastic modulus
